# Effect of a serogroup A meningococcal conjugate vaccine (PsA–TT) on serogroup A meningococcal meningitis and carriage in Chad: a community study

**DOI:** 10.1016/S0140-6736(13)61612-8

**Published:** 2013-01-04

**Authors:** DM Daugla, JP Gami, K Gamougam, N Naibei, L Mbainadji, M Narbé, J Toralta, B Kodbesse, C Ngadoua, ME Coldiron, F Fermon, A-L Page, MH Djingarey, S Hugonnet, OB Harrison, LS Rebbetts, Y Tekletsion, ER Watkins, D Hill, DA Caugant, D Chandramohan, M Hassan-King, O Manigart, M Nascimento, A Woukeu, C Trotter, JM Stuart, MCJ Maiden, BM Greenwood

**Affiliations:** a Centre de Support en Santé International (CSSI), N'Djamena, Chad; b Ministry of Public Health, N'Djamena, Chad; c Epicentre, Médecins sans Frontères, Paris, France; d WHO Intercountry Support Team, Ougadougou, Burkina Faso; e Department of Pandemic and Epidemic Diseases, WHO, Geneva, Switzerland; f Department of Zoology, University of Oxford, Oxford, UK; g Norwegian Institute for Public Health, Oslo, Norway; h Department of Veterinary Medicine, University of Cambridge, Cambridge, UK; i Faculty of Infectious and Tropical Diseases, London School of Hygiene and Tropical Medicine, London, UK

## Abstract

**Background:**

A serogroup A meningococcal polysaccharide–tetanus toxoid conjugate vaccine (PsA–TT, MenAfriVac) was licensed in India in 2009, and pre-qualified by WHO in 2010, on the basis of its safety and immunogenicity. This vaccine is now being deployed across the African meningitis belt. We studied the effect of PsA–TT on meningococcal meningitis and carriage in Chad during a serogroup A meningococcal meningitis epidemic.

**Methods:**

We obtained data for the incidence of meningitis before and after vaccination from national records between January, 2009, and June, 2012. In 2012, surveillance was enhanced in regions where vaccination with PsA–TT had been undertaken in 2011, and in one district where a reactive vaccination campaign in response to an outbreak of meningitis was undertaken. Meningococcal carriage was studied in an age-stratified sample of residents aged 1–29 years of a rural area roughly 13–15 and 2–4 months before and 4–6 months after vaccination. Meningococci obtained from cerebrospinal fluid or oropharyngeal swabs were characterised by conventional microbiological and molecular methods.

**Findings:**

Roughly 1·8 million individuals aged 1–29 years received one dose of PsA–TT during a vaccination campaign in three regions of Chad in and around the capital N'Djamena during 10 days in December, 2011. The incidence of meningitis during the 2012 meningitis season in these three regions was 2·48 per 100 000 (57 cases in the 2·3 million population), whereas in regions without mass vaccination, incidence was 43·8 per 100 000 (3809 cases per 8·7 million population), a 94% difference in crude incidence (p<0·0001), and an incidence rate ratio of 0·096 (95% CI 0·046–0·198). Despite enhanced surveillance, no case of serogroup A meningococcal meningitis was reported in the three vaccinated regions. 32 serogroup A carriers were identified in 4278 age-stratified individuals (0·75%) living in a rural area near the capital 2–4 months before vaccination, whereas only one serogroup A meningococcus was isolated in 5001 people living in the same community 4–6 months after vaccination (adjusted odds ratio 0·019, 95% CI 0·002–0·138; p<0·0001).

**Interpretation:**

PSA–TT was highly effective at prevention of serogroup A invasive meningococcal disease and carriage in Chad. How long this protection will persist needs to be established.

**Funding:**

The Bill & Melinda Gates Foundation, the Wellcome Trust, and Médecins Sans Frontères.

## Introduction

For more than 100 years, the Sahelian and sub-Sahelian regions of Africa have had periodic, large, and unpredictable epidemics of meningococcal meningitis.^1^ The first outbreaks in Chad were reported in 1916 and 1918,^2^ and major epidemics arose in 1924 and 1935–39, with a mortality rate of more than 75% in both epidemics.^3^ Another major cycle began in 1943, when more than 2000 cases were reported, and further epidemics arose in the 1950s and 1960s. In 1968, an outbreak including 272 cases was reported in the capital N'Djamena (formerly Fort Lamy), but mortality was only 10% after introduction of treatment with sulphonamides, penicillin, or chloramphenicol.^4^ A large epidemic in the Logone Occidental region in 1988, and an outbreak in the Goundi district in 2001, showed the characteristic seasonality of epidemic meningitis in Africa.^5^, ^6^ A new, nationwide epidemic in 2009–12 included 1000 cases.^7^ Serogroup A meningococci were present in all early outbreaks in which the epidemic strain was characterised.^8^, ^9^ However, serogroup W meningococci were isolated from 2005 onwards, and both serogroup A and serogroup W infections were detected during the 2009 epidemic, with the preponderant serogroup varying between areas.^10^

Chad was one of the first countries in Africa to attempt to prevent meningococcal disease by vaccination. In 1936, a serogroup A whole cell vaccine was produced at Sarh (formerly Fort Archambault) and widely distributed throughout Chad with apparent success, although no clinical trial was done.^11^ Polysaccharide vaccines were first used extensively in the 1988 epidemic. An initial campaign in which vaccination was restricted to schoolchildren and the military had little or no effect on the course of the epidemic. A second universal vaccination campaign might have been more effective, but vaccination was introduced near the end of the season when the epidemic was expected to decrease.^12^ Sarh was included in this campaign, but a major outbreak happened in the town 2 years later,^13^ and epidemics have continued in Chad despite widespread use of polysaccharide vaccines in reactive vaccination campaigns.

Polysaccharide–protein conjugate vaccines are more likely to prevent epidemics than are polysaccharide vaccines because conjugate vaccines are immunogenic in young children, induce immunological memory, and prevent pharyngeal carriage.^14^ Because most of the meningococcal epidemics in Africa have been caused by serogroup A meningococci, the Meningitis Vaccine Project (MVP), a public–private partnership between WHO and PATH, was established in 2001, with support from the Bill & Melinda Gates Foundation, to develop an affordable, monovalent serogroup A conjugate vaccine. As a result of an innovative and successful north–south development programme, a serogroup A polysaccharide–tetanus toxoid conjugate vaccine (PsA–TT, MenAfriVac) was developed at the Serum Institute of India (Pune, India). This vaccine was licensed in India in 2009, and pre-qualified by WHO in 2010, on the basis of the safety and high immunogenicity of the vaccine;^15^ no phase 3 efficacy trial was undertaken. Thus, assessment of the efficacy of PsA–TT after licensure is essential. Roll-out of PsA–TT, supported by the MVP, WHO, UNICEF, and GAVI Alliance, started in December 2010, when nearly all 1–29 year-olds in Burkina Faso were vaccinated during 6 weeks.^16^ In the year after this campaign, the incidence of overall cases of meningitis in Burkina Faso fell to the lowest rate recorded since 1995, no local outbreaks were reported, and no case of serogroup A disease was detected.^17^ At the same time, a marked drop in the prevalence of serogroup A meningococcal carriage was noted in three districts in which carriage surveys were done.^18^ These results strongly suggested a major effect of PsA–TT on serogroup A meningococcal disease and carriage, but they cannot be regarded as definitive because the vaccine was introduced at a time of very low and decreasing transmission of the serogroup A meningococcus in the central part of the African meningitis belt. In 2011, Chad and northern Cameroon were the only parts of the African meningitis belt where serogroup A cases were detected in substantial numbers.^19^

The African Meningococcal Carriage Consortium (MenAfriCar) was established to study the epidemiology of meningococcal carriage in countries of the African meningitis belt before and after the introduction of PsA–TT.^20^ MenAfriCar started work in Chad in 2009 and was, therefore, able to measure the effect of PsA–TT on meningitis and meningococcal carriage in this country after the first phase of mass vaccination in 2011.

## Methods

### Study area

Chad is one of the largest countries in Africa with a surface area greater than 1 million km^2^. The north of the country is desert, the centre is arid Sahel, and the south a more fertile Sudan Savanna zone. The central and southern parts of the country have a typical Sahelian climate with a short rainy season, maximum in the south, and a long dry season during which epidemics of meningitis can happen. The population, roughly 11 million, is concentrated in the southern part of the country. Health care is provided through about 800 health centres, 60 district hospitals, ten regional hospitals, and two tertiary hospitals in N'Djamena.

### Surveillance for meningitis

**Figure 1:**
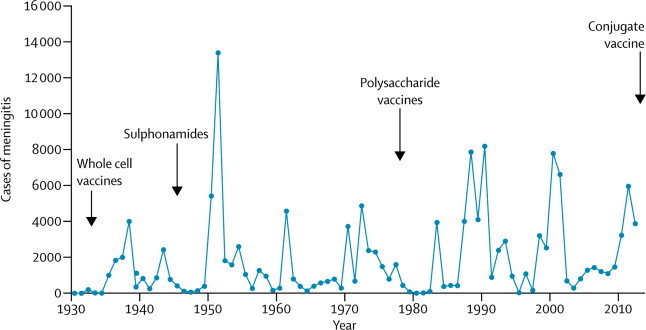
Reported cases of meningitis in Chad from 1930–2012 Arrows show the introduction of new control measures.

Clinically diagnosed cases and deaths due to meningitis are recorded at all health centres and district hospitals in Chad and these aggregated data are transmitted every week to the district medical officer. Data from each district are submitted to the integrated epidemiological service of the Ministry for Public Health, which passes this information on to the WHO Inter-Country Support Team in Ouagadougou, Burkina Faso (figure 1). Cerebrospinal fluid (CSF) samples are collected, transferred to the central hospital in N'Djamena, and tested by routine microbiology or by latex test; the latex test is also being used in the field. The completeness of case ascertainment is unknown. From March 1, 2012, to June 30, 2012, surveillance was enhanced, with support from the MenAfriCar consortium, in the three regions (N'Djamena, Chari-Baguirmi, and Mayo-Kebbi Est) in which individuals aged 1–29 years (target population 1·8 million) had been vaccinated with PsA–TT during 10 days in December, 2011, shortly before the 2012 epidemic season. A nurse and a laboratory technician were identified at each hospital and given responsibility to complete a case report form on any suspected case of meningitis and to ensure that CSF samples were transported to the national reference laboratory in a plain tube and in trans-isolate medium. Additionally, case surveillance was established during an outbreak from March 4, to May 5, 2012, in one district (Moissala) that had not received PsA–TT and where reactive vaccination was undertaken.

### Carriage studies

Between September and November, 2010, we did a carriage survey in 998 age-stratified residents of the rural area of Mandelia, roughly 65 km south of N'Djamena. Between August and October, 2011, we did a pre-vaccination survey in 4278 age-stratified individuals; between April and June, 2012, a survey was done after vaccination in 5001 individuals in the same community, sampled so that four age groups (0–4 years, 5–14 years, 15–29 years, and >30 years) were adequately represented. A higher proportion of school age children and young adults were included in the post-vaccination survey to increase the likelihood of detection of serogroup A carriers. Details of the sampling methods and swabbing techniques used are described elsewhere.^20^

### Laboratory methods

CSF samples were gram stained, tested for microbial antigens (Pastorex test, Bio-Rad Laboratories, Marnes-la-Coquette, France), and cultured on chocolate agar or Thayer-Martin agar (Oxoid SR0091E, Oxoid Ltd, Basingstoke, UK]) with antibiotic supplements (3 mg/L vancomycin, 7·5 mg/L colistin, 1250 U/L nystatin, and 5 mg/L trimethoprim) with incubation in 5–10% carbon dioxide. Meningococci identified by morphology and biochemical tests (API NH or VITEK2 Compact, Biomérieux, Marcy l'Etoile, France) were serogrouped by agglutination, and the antimicrobial sensitivity of meningococci was established. *PorA* and genogrouping PCR were done on specimens from the area where reinforced surveillance had been introduced in 2012. Strains isolated from cases of meningitis were stored in brain infusion broth with glycerol at −80°C and sent to the Inter-Country Support Team, Ouagadougou, and to the WHO Collaborating Centre for Reference and Research on Meningococci in Oslo, Norway. CSF samples collected in trans-isolate medium from outbreak areas by a team from Médecins Sans Frontères (MSF) were analysed by genogrouping and *PorA* PCR in Oslo.

The laboratory methods used to detect meningococcal carriage are described in detail elsewhere.^20^ In brief, swabs were plated directly onto a modified Thayer-Martin plate containing antibiotics and transferred to the laboratory within 6 h of collection. Colonies with a morphology characteristic of *Neisseria* species were subcultured onto two blood agar plates. Isolates that were γ-glutamyl transpeptidase (GGT) positive, o-nitrophenyl-β-d-galactopyranoside (ONPG) negative, and tributyrin negative were characterised as presumptive *N meningitidis* and serogrouped by slide agglutination. Heat-killed cell suspensions were prepared from all isolates characterised as oxidase-positive, Gram-negative diplococci and sent to the Department of Zoology, University of Oxford, Oxford, UK, for molecular confirmation of speciation, genogroup, and *porA* genosubtype. *Neisseria* species were confirmed by sequencing a 413 bp fragment of the *rplF* ribosomal subunit gene,^21^ and samples not confirmed as *Neisseria* were speciated by sequencing the 16S rRNA gene.^22^ The capsule region was characterised by sequencing to identify the capsule null (*cnl*) region^23^ and a real-time PCR assay was used to detect genes encoding serogroup A, W, and X capsular polysaccharides.^24^ Sequencing of the *porA* gene confirmed likely membership of the sequence type 5 (ST-5) clonal complex (formerly known as subgroup 3),^25^ which has caused serogroup A epidemics in the meningitis belt since the Hajj epidemics of the late 1980s.^26^

### Statistical analysis

We used data from the 2009 census as the denominator to calculate incidence rates. To assess the effect of PsA–TT on the incidence of meningitis, we analysed weekly incidence data collected during the 26 weeks of active surveillance each year from Jan 1, 2009, to June 30, 2012, from two areas, one of which was vaccinated in 2012 (area one), and the other remained unvaccinated (area two). We measured the effect of vaccination in two ways with a negative binomial regression model. First, we assessed the crude difference in total incidence in 2012 between vaccinated and unvaccinated areas. Then, to estimate the effect of vaccination above the difference between the areas in 2009–11, we incorporated an interaction term for area one in 2012.

To assess the effect of PsA–TT on carriage of serogroup A meningococci, we compared the prevalence of carriage before and after vaccination in Mandelia (rural area) with a logistic regression model adjusted for age. This adjustment was necessary because the sampling strategy had been modified between the 2011 and 2012 surveys, with the 2012 survey sampling fewer younger children who were shown in 2011 to rarely carry meningococci. All analyses were done with Stata (version 12.0). We estimated vaccine coverage in the targeted group aged 1–29 years as the total number vaccinated divided by the population size in 2009. The study is registered with ClinicalTrials.gov, number NCT01119482.

### Role of the funding sources

The sponsors of the study had no role in study design, data collection, data analysis, data interpretation, or writing of the report. The corresponding author had full access to all the data in the study and had final responsibility for the decision to submit for publication.

## Results

**Figure 2:**
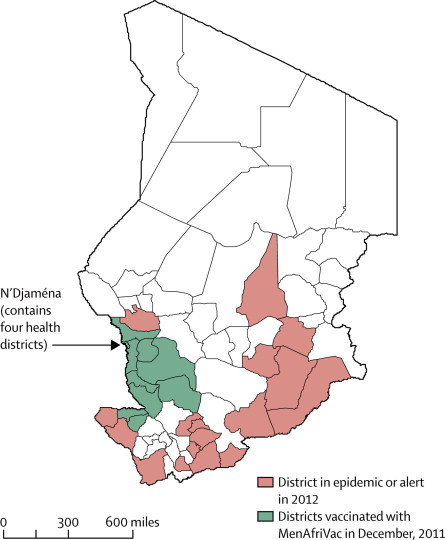
Areas of Chad vaccinated with PsA–TT in 2011, or in epidemic or alert situation during Jan–June 2012 Green shading shows the districts where mass vaccination of people aged 1–29 years was undertaken at the end of 2011, before the 2012 meningitis season. Red shading shows the districts where the alert or epidemic threshold was reached during the course of the 2012 meningitis season. PsA–TT=serogroup A meningococcal polysaccharide–tetanus toxoid conjugate vaccine.

The present epidemic of meningitis in Chad began in 2009 with an upsurge in cases of both serogroup A and serogroup W meningococcal meningitis. 3058 cases were reported in 2010, 5960 in 2011, and 3795 in 2012 (figure 2). During 2011 and 2012, most meningococcal isolates obtained were serogroup A (data not shown). In the three regions in which vaccination was undertaken in December, 2011, estimated vaccine coverage was 102%. These regions were chosen for logistical reasons. Nationwide vaccination of the 1–29-year-old population was achieved in three further phases between June and December, 2012, with estimated vaccine coverage of 95%, 95%, and 81%, respectively. Coverage in the last phase of the vaccination campaign fell after reports of adverse events after vaccination, concerns that were subsequently shown to be unfounded.^27^

Reactive vaccination campaigns with serogroups A and C or serogroups A,C, and W plain polysaccharide vaccines were implemented in response to outbreaks in nine districts in 2009 (A+C), in eight districts in 2010 (A+C [three]; A+C+W [five]), and in 12 districts and the refugee camp of Tréguine (district of Adré) in 2011 (A+C). Between February and May, 2012, reactive vaccination campaigns with the PsA–TT conjugate vaccine were done in nine districts that had passed the epidemic threshold and in three other districts next to epidemic districts. Vaccine coverage with PsA–TT in the target population (1·5 million) during the 2012 reactive vaccination campaigns was estimated to be 94%. In five of the 12 districts that received PsA–TT in 2011, reactive vaccination with polysaccharide serogroups A and C vaccine had been done in 2009. Reactive vaccination campaigns with polysaccharide vaccines were not undertaken in any of these 12 districts in 2010, 2011, or 2012.

**Figure 3:**
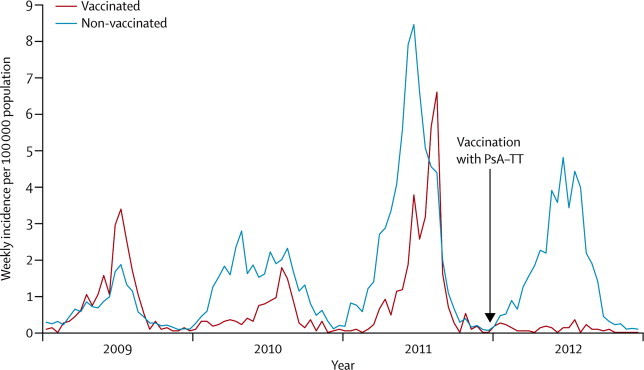
Incidence of reported cases of meningitis in Chad, 2009–12 Vaccination with PsA–TT was undertaken in patients aged 1–29 years at the end of 2011 (arrow). PsA–TT=serogroup A meningococcal polysaccharide–tetanus toxoid conjugate vaccine.

The incidence of reported cases of meningitis during the first 26 weeks of 2012 in the three regions where vaccination with PsA–TT of individuals aged 1–29 years had been undertaken the previous year was 2·48 per 100 000 (57 cases per 2·3 million population). By contrast, the incidence in areas where PsA–TT vaccination had not been undertaken as part of the mass campaign, including the areas where reactive vaccination was undertaken in response to an outbreak, was 43·8 per 100 000 (3809 cases per 8·7 million population), a 94% difference in total incidence in 2012 (p<0·0001). 17 districts reached the alert or epidemic threshold in 2012, none of which were in the three vaccinated regions (figure 2). The difference in incidence between vaccinated and unvaccinated regions recorded in 2012 showed a marked change from the pattern recorded in the previous 3 years (figure 3). Results from a negative binomial regression model suggest a 90·4% (p<0·0001) overall reduction in the risk of meningitis after mass vaccination, with an estimated incidence rate ratio of 0·096 (95% CI 0·046–0·198).

In 2012, laboratory confirmation of a diagnosis of meningococcal meningitis was made in 65 of 366 CSF samples (18%) submitted to the national reference laboratory in N'Djamena. A diagnosis of serogroup A meningococcal meningitis was made by culture or latex test in 59 of 329 samples from people in regions where vaccination had not been done previously with PsA–TT. Serogroup A meningococcus was not isolated from any of the 37 samples from the three vaccinated regions with enhanced surveillance (table 1), even though only individuals aged 1–29 years had been vaccinated. Ten serogroup A isolates obtained from cases of meningitis studied at the National Institute for Public Health, Oslo, Norway, were all characterised as belonging to ST-7 of the ST-5 clonal complex, subtype P1·20,9.

**Table 1:** Diagnosis of serogroup A meningococcal meningitis in individuals from non-vaccinated and vaccinated regions in Chad

	**Number of CSF samples**	Diagnosis*					
		**Meningococci**				**Pneumococci**	**Other**
		A	W	X	Other		
**Non-vaccinated regions**†							
1–29 years	305	57	3	0	0	2	0
<1 or ≥30 years	24	2	1	0	0	2	0
Total	329	59	4	0	0	4	0
**Vaccinated regions**							
1–29 years	34	0	0	2	0	0	0
<1 or ≥30 years‡	3	0	0	0	0	0	0
Total	37	0	0	2	0	0	0

CSF=cerebrospinal fluid.

* Diagnosis based on culture or latex test.

† Includes regions where reactive vaccination in response to an outbreak was undertaken.

‡ Not in the target group for vaccination.

The incidence of meningitis reached the epidemic threshold in weeks 6–22 in 12 districts, initiating reactive vaccination campaigns undertaken by the Ministry of Health, WHO, MSF, and other agencies. The effect of reactive vaccination was studied in detail in one district (Moissala, attack rate 148 per 100 000 population, case fatality rate 3·4%) where CSF samples were obtained from 176 of 334 (53%) reported cases. Before vaccination with PsA–TT, 48 serogroup A infections were detected by latex agglutination and 35 by culture or PCR; the data for serogroup W were 18 and 17, respectively. After the vaccination campaign, only one further case of serogroup A meningitis was detected in a 9-year-old girl who had been vaccinated 5 days previously (appendix).

The prevalence of meningococcal carriage in the rural area of Mandelia varied with age, and was most frequent in individuals aged 1–29 years. The overall prevalence of meningococcal carriage was low in two surveys undertaken before, and one survey undertaken after vaccination of individuals aged 1–29 years with PsA–TT (table 2). Serogroup A carriers were defined according to the characteristics of their pharyngeal isolate—namely, whether the isolate provided DNA corresponding to either serogroup A capsule region, *rplF* allele 1 (*rplF-1*) and a *porA* gene encoding the subtype P1·20,9 (criterion one); or to a serogroup A capsular gene alone (criterion two). The first definition was regarded as confirmation that the individual was carrying an epidemic strain—ie, a serogroup A, ST-5 clonal complex meningococcus. Most of the individuals with serogroup A capsular DNA were probably also carrying such a strain, perhaps at a low density. 2–4 months before vaccination, 32 of 4278 individuals (0·75%) met criterion one and an additional 12 met criterion two. 4–6 months after the vaccination campaign, only one of 5001 individuals tested (0·02%) met criterion one, a 98% difference in prevalence (adjusted odds ratio 0·019, 95% CI 0·002–0·138), and no additional individual met criterion two. The one serogroup A carrier detected after vaccination was a 15-year-old boy who, according to his vaccination card, had been vaccinated with PsA–TT 4 months before detection. The number of individuals carrying serogroup A fell in the unvaccinated age groups from seven of 1374 before vaccination to zero of 336 individuals after vaccination (p=0·19).

**Table 2:** Meningococcal carriage in the Mandelia district of Chad before and after vaccination with PsA–TT

	**Number tested**	**Meningococci isolated**				
		All	A, P1·20,9*	W	X	Other†
**Survey one (September–November, 2010, 13–15 months before vaccination)**						
<1 year	74	0	0	0	0	0
1–29 years	670	6	6	0	0	0
≥30 years	254	0	0	0	0	0
Total	998	6	6	0	0	0
Carriage prevalence	..	0·6%	0·6%	0%	0%	0%
**Survey two (August–October, 2011, 2–4 months before vaccination)**						
<1 year	260	1	1	0	0	0
1–29 years	2904	45	25‡	3	6	11
≥30 years	1114	10	6	0	0	4
Total	4278	56	32	3	6	15
Carriage prevalence	..	1·3%	0·7%	0·07%	0·1%	0·4%
**Survey three (April–June, 2012, 4–6 months after vaccination)**						
<1 year	68	0	0	0	0	0
1–29 years	4665	39	1	1	5	32
≥30 years	268	2	0	0	0	2
Total	5001	41	1	1	5	34
Carriage prevalence	..	0·8%	0·02%	0·02%	0·1%	0·7%

PsA–TT=serogroup A meningococcal polysaccharide–tetanus toxoid conjugate vaccine.

* Specimens in which the meningococcal serogroup A capsular region, *rplF* allele 1 (*rplF-1*), and a *porA* gene subtypes P1·20,9 were detected.

† Other serogroups include specimens that were confirmed as *Neisseria meningitidis*, but not characterised as serogroup A subtype P1·20,9 or serogroup X or W. Further inf ormation about these specimens are given in the appendix.

‡ One of these specimens yielded both a serogroup A and a serogroup X meningococcus.

## Discussion

This study has established the ability of a new serogroup A meningococcal conjugate vaccine (PsA–TT) to prevent epidemic meningitis in Chad, a country within the African meningitis belt where epidemics are frequent and severe. The vaccine halted a continuing epidemic in districts around the capital N'Djamena while the epidemic continued in other parts of the country. Vaccination was associated with a marked drop in pharyngeal carriage of the serogroup A epidemic strain, and this probably contributed to the substantial effect of the vaccine.

Measurement of the effectiveness of a vaccine after its introduction is especially important when licensure has been based solely on safety and immunogenicity, as has been the case for PsA–TT and other conjugate vaccines. Studies done before and after vaccination can provide some evidence for an effect,^28^ but can be affected by temporal changes in disease incidence that are independent of any intervention. This constraint applies especially to infections that tend to be epidemic such as meningococcal infection. The fact that only part of Chad was vaccinated at the end of 2011 provided a unique opportunity to measure the effect of PsA–TT on serogroup A meningococcal disease by measurement of disease incidence in vaccinated and unvaccinated areas at the same time.

At the end of 2011, shortly before the 2012 epidemic season, vaccination of individuals aged 1–29 years (target population 1·8 million) was undertaken in three regions of Chad. The estimated coverage of the mass campaign was 102%. This apparently anomalous figure might be indicative of inaccuracies in the census data used as a denominator because the census was done 3 years before vaccination. Other possible explanations could be that individuals older than the targeted limit of 29 years were vaccinated, people travelled to the region to be immunised, or some individuals were vaccinated twice.

During the 2012 meningitis season, the incidence of meningitis fell substantially in areas of Chad where individuals aged 1–29 years had been vaccinated with PsA–TT a few months previously while the epidemic continued in neighbouring non-vaccinated areas. The disparity between vaccinated and non-vaccinated groups was probably even greater than we have reported here for two reasons. First, surveillance for cases of meningitis was enhanced in the vaccinated areas but, for financial and logistical reasons, not in the rest of the country where reporting of cases was dependent upon a less rigorous routine surveillance system. Despite enhanced surveillance, no case of serogroup A meningitis was detected in a population of roughly 2 million residents in the vaccinated areas. Second, reactive vaccination was undertaken in the middle of the 2012 meningitis season in 12 districts that had passed the epidemic threshold. Had reactive vaccination not been undertaken in these districts, more cases would probably have been recorded, increasing the disparity in incidence between unvaccinated and vaccinated areas.

Because vaccinated and non-vaccinated districts were not randomised, the difference in incidence of meningitis between vaccinated and non-vaccinated areas could have been caused by random fluctuations in disease incidence. However, we think that this situation is very unlikely for two main reasons. First, surveillance data suggest that the epidemic was progressing in a similar way in vaccinated and in unvaccinated areas before vaccination (figure 3), and second, the geographical distribution of cases in the 2012 outbreak (figure 1) suggests that the vaccinated areas where no cases were recorded are surrounded by areas where the epidemic or alert threshold was reached.

The drop in the number of cases of meningitis recorded in vaccinated areas was accompanied by a 98% decrease in the prevalence of serogroup A meningococcal carriage in all age groups in a rural area where the serogroup A meningococcal carriage rate had been about 1% before vaccination with PsA–TT. This decrease was equally large when the definition of carriage was detection of the serogroup A epidemic strain or the likely presence of a serogroup A meningococcus. For logistical and financial reasons, carriage studies in non-vaccinated areas during the period in which post-vaccination carriage findings were made in the vaccinated population were not possible. However, the fact that the incidence of serogroup A disease remained high in these non-vaccinated areas suggests that transmission of the serogroup A meningococcus was continuing in these districts and that carriers were present.

No cases of serogroup A meningitis were detected in residents of the vaccinated areas who were too young or too old to be vaccinated. Only eight cases of meningitis of any kind were reported in this population, although about 100 would have been expected in view of the overall attack rate in the unvaccinated population and the age distribution of cases detected in the unvaccinated Moissala district. These findings, together with the absence of any serogroup A meningococcal carriage in the unvaccinated population in the areas where mass vaccination had been undertaken, suggest that PsA–TT has an important, indirect effect on serogroup A carriage and disease, in keeping with the results of studies in Burkina Faso (panel).^17^, ^18^ The result of studies done before and after intervention in Burkina Faso suggested that PsA–TT had a major effect on both the incidence of meningitis and of serogroup A carriage in that country,^17^, ^18^ but the incidence of serogroup A disease in Burkina Faso was falling before the introduction of the vaccine and so the further decrease recorded in 2011 could have been caused by naturally acquired immunity to the epidemic clone. Because national vaccination coverage was achieved within a short period in Burkina Faso, the comparison of data from vaccinated and non-vaccinated areas was not possible. By contrast, in Chad, we were able to describe the incidence of meningitis in vaccinated and unvaccinated areas over the same period during an epidemic.

Panel **Research in context**

**Systematic review**

We searched PubMed with the search terms “Africa” AND (“Neisseria” OR “meningococci”) AND “vaccine” AND “conjugate” AND (“evaluation” OR “impact” OR “effectiveness” OR “uptake” OR “coverage”), to identify papers published between Jan 1, 2010, and May 1, 2013, because 2010 was the first year that serogroup A meningococcal polysaccharide–tetanus toxoid conjugate vaccine (PsA–TT, MenAfriVac) was used after phase 1 and 2 clinical trials. Five of the 27 papers retrieved were relevant to understanding the effect of population-based vaccination campaigns with PsA–TT. Three studies reported high vaccine uptake in Burkina Faso^29^ and Niger.^30^, ^31^ Two papers from Burkina Faso reported the effect of PsA–TT introduction on rates of meningitis^18^ and meningococcal carriage.^19^ In the study of meningitis rates, which analysed meningitis incidence rates from surveillance data, a mass vaccination campaigns in people aged 1–29 years was estimated to reduce the risk of meningitis by 71% overall, whereas the risk of disease caused by laboratory-confirmed serogroup A *Neisseria meningitidis* fell by 99·8%. However, the incidence of serogroup A meningitis was low at the time of vaccine introduction in this country. The carriage surveys in Burkina Faso did not identify any serogroup A carriers after vaccination, compared with a baseline prevalence of 0·39%.

**Interpretation**

Our study is the first to measure the effect of PsA–TT on meningococcal disease and carriage in a country with an epidemic of serogroup A meningitis at the time of vaccine introduction. The incidence of meningitis fell substantially in vaccinated areas whereas the epidemic continued in unvaccinated parts of the country. PsA–TT seems to be highly effective against both serogroup A meningococcal disease and carriage. Questions remain about the duration of protection provided by this vaccine and whether elimination of the serogroup A meningococcus will be followed by an upsurge in cases caused by meningococci belonging to other serogroups. To answer these questions will need continued surveillance for meningitis and further carriage studies in countries of the African meningitis belt.

Data from studies done in Chad strongly suggest that PsA–TT has a major effect on serogroup A meningococcal disease and carriage and support the continuing roll-out of this vaccine across the African meningitis belt. However, several more years of active surveillance are needed to establish the duration of protection provided by PsA–TT, whether the vaccine can prevent future epidemics, and whether reduction of serogroup A meningococcal carriage will lead to an increase in carriage with other meningococci.^32^ Replacement could be beneficial if the strain that replaces the serogroup A meningococcus is non-pathogenic, or it could be a major concern for future vaccination strategies if the replacement strain is able to cause epidemics.

**This online publication has been corrected twice. The first corrected version appeared at thelancet.com on October 25, 2013, and the second on January 3, 2014**

## Supplementary Material

Supplementary video The effects of PsA-TT vaccine on meningococcal meningitis Chad during a serogroup A meningococcal meningitis epidemic.
