# Supplementary material for: Effect of a serogroup A meningococcal conjugate vaccine (PsA–TT) on serogroup A meningococcal meningitis and carriage in Chad: a community study
Source: Lancet. 2013 Jan 4;383(9911):40–7. doi: 10.1016/S0140-6736(13)61612-8 (PMC3898950; doi:10.1016/S0140-6736(13)61612-8)
Supplement: Supplementary appendix [file mmc2.pdf]

THE LANCET

Supplementary appendix

This appendix formed part of the original submission and has been peer reviewed. We post it as supplied by the authors.

Supplement to: Daugla DM, Gami JP, Gamougam K, et al. Effect of a serogroup A meningococcal conjugate vaccine (PsA–TT) on serogroup A meningococcal meningitis and carriage in Chad: a community trial. *Lancet* 2013; published online Sept 12. http://dx.doi.org/10.1016/S0140-6736(13)61612-8.

SUPPLEMENTARY INFORMATION

1. Reactive vaccination with PsA-TT in epidemic districts.

In twelve districts of Chad where the epidemic threshold was reached, reactive vaccination of subjects aged 1-29 years was undertaken by a team from the Ministry of Health supported by Médecins sans Frontières and other voluntary agencies. In the district of Moissala, where surveillance was enhanced, cases declined following vaccination (Supplementary Figure 1) but had begun to do so before vaccination and the outbreak was probably already in decline.

2. Characteristics of meningococcal carriage isolates obtained from Chad

DNA samples obtained from Oxidase-positive, Gram negative bacteria collected during pre- and post-vaccination surveys conducted in Chad were transferred to the Department of Zoology, the University of Oxford where the identity of these bacteria was characterised further using the methods described in the main paper. *Neisseria* species was confirmed by sequencing a 413 bp fragment of the *rplF* ribosomal subunit gene,[1] and samples not confirmed as *Neisseria* were speciated by sequencing the 16S rRNA gene.[2] The capsule region was characterised by sequencing to identify the capsule null (*cnl*) region [3] and a real-time PCR assay was employed to detect genes encoding serogroup A, W, and X capsular polysaccharides.[4] Sequencing of the *porA* gene confirmed likely membership of the ST-5 complex (formerly known as subgroup III) [5] which has been responsible for serogroup A epidemics in the meningitis belt since the Hajj epidemics of the late 1980s.[6] The result of speciation, genogrouping and outer membrane protein characterisation are shown in supplementary table 1. In some cases the capsular gene of a serogroup A meningoccus was detected in the presence of another bacterium, probably a result of the presence of mixed carriage infections.

References.

1. Bennett JS, Jolley KA, Earle SG, et al. A genomic approach to bacterial taxonomy: an examination and proposed reclassification of species within the genus *Neisseria*. *Microbiology* 2012; **158**: 1570-80.
2. Harmsen D, Singer C, Rothgänger J, et al. Diagnostics of neisseriaceae and moraxellaceae by ribosomal DNA sequencing: ribosomal differentiation of medical microorganisms. *J Clin Microbiol* 2001; **39**: 936-42.
3. Claus H, Maiden MC, Maag R, Frosch M, Vogel U. Many carried meningococci lack the genes required for capsule synthesis and transport. *Microbiology* 2002; **148**: 1813-19.
4. Wang X, Theodore MJ, Mair R, et al. Clinical validation of multiplex real-time PCR assays for detection of bacterial meningitis pathogens. *J Clin Microbiol* 2012; **50**: 702-08.
5. Suker J, Feavers IM, Achtman M, Morelli G, Wang JF, Maiden MC. The porA gene in serogroup A meningococci: evolutionary stability and mechanism of genetic variation. *Mol Microbiol* 1994; **12**: 253-65.
6. Caugant DA, Maiden MC. Meningococcal carriage and disease-population biology and evolution. *Vaccine* 2009; **27 Suppl 2**: B64-70.

Supplementary Figure 1. Cases of meningitis by week of admission Moissala district, Chad, 2012 a) all probable and confirmed cases (n = 334) (b) confirmed cases by week and serogroup (n =79)*

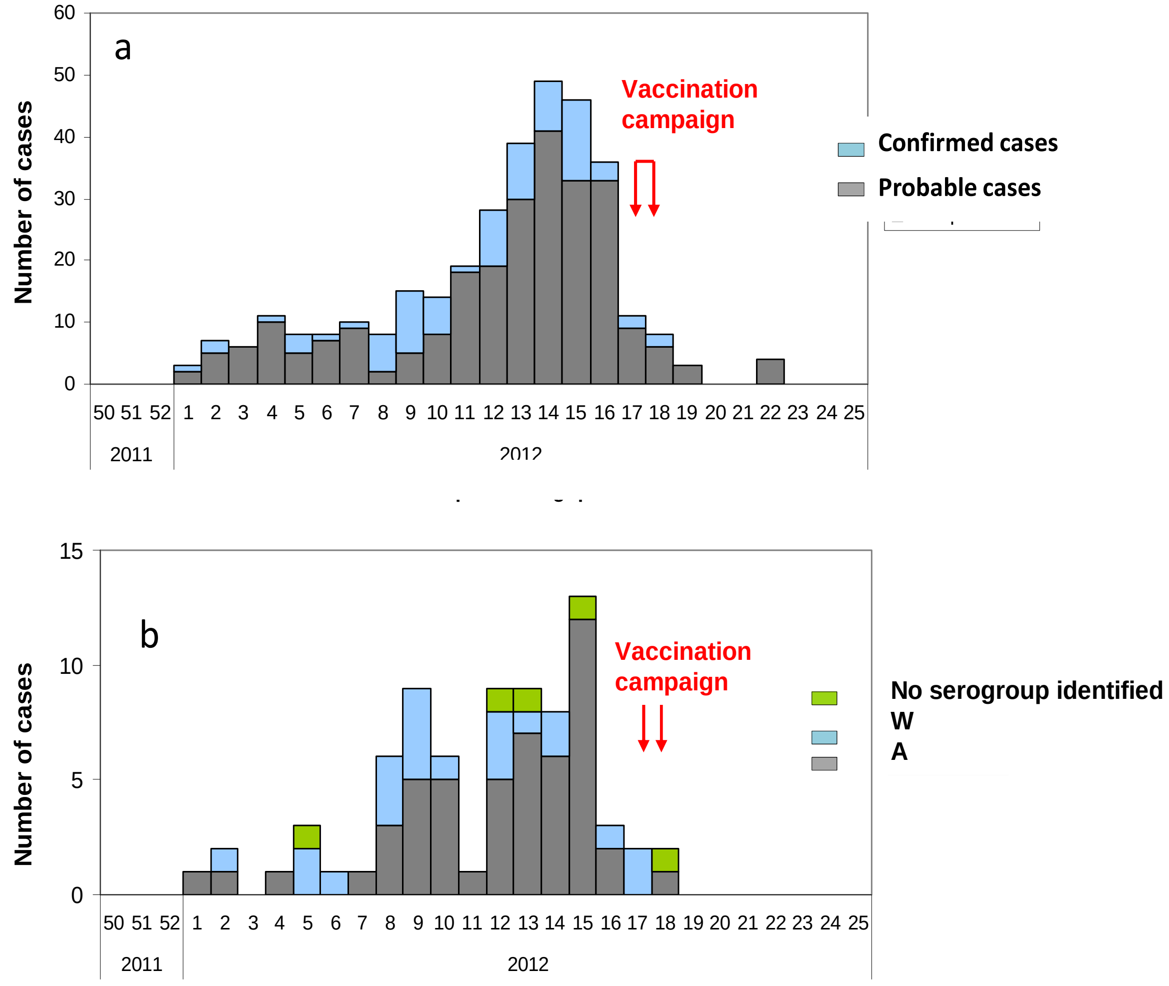

***One confirmed case of group B *Neisseria meningitidis* that occurred in week 9 is not included in these figures.**

Supplementary Table 1. Detailed molecular characteristics of the carriage isolates undertaken at the department of Zoology, University of Oxford.

Species	Capsular group	PorA	Frequency Pre-vaccination survey	Frequency Post-vaccination survey
Neisseria meningitidis	A	P1.20, 9	32	1
Neisseria meningitidis	A	none identified	3	0
Other*	A	none identified	9	0
Neisseria meningitidis	W	P1.5,2	2	0
Neisseria meningitidis	W	P1.5-1, 2-36	1	1
Neisseria meningitidis	X	P1.5-1, 10-1	6	5
Other*	X	none identified	0	1
Neisseria meningitidis	cnl	P1.18-11, 42-2	3	22
Neisseria meningitidis	cnl	P1.18-11, -	0	1
Neisseria meningitidis	cnl	P1.18, 25-19	1	0
Neisseria meningitidis	cnl	P1.18, 25	0	1
Neisseria meningitidis	cnl	P1.19, 15-15	0	1
Neisseria meningitidis	cnl	P1.22-11, -	1	2
Neisseria meningitidis	cnl	P1.22, 14-6	1	0
Neisseria meningitidis	cnl	P1.21-14, 28-3	0	4
Neisseria meningitidis	cnl	P1.21-14, -	0	1
Neisseria meningitidis	cnl	P1.22-11, 15-25	0	1
Neisseria meningitidis	cnl	none identified	0	1
Neisseria meningitidis	none identified	P1.18-11, 42-2	1	0
Neisseria meningitidis	none identified	P1.22-11, 15-25	3	0
Neisseria meningitidis	none identified	P1.22-1, 14	1	0
Neisseria meningitidis	none identified	P1,21-14, 28-3	1	0

* Includes not identified

Total *Neisseria meningitidis*	56	41
Prevalence of meningococcal carriage	1.3%	0.8%
Additional samples with capsular genes	9	1
